# Effect of Simmering Technology on Components and Activity of *Myristica fragrans* Houtt.

**DOI:** 10.3390/molecules28227627

**Published:** 2023-11-16

**Authors:** Jiaqi Sun, Fuyin Zhao, Zhenlei Wang, Weina Zhang, Xiudong Yang, Hongli Zhou, Peng Wan

**Affiliations:** 1 Department of Physiology, Jilin Medical University, Jilin 132013, China; 18686299758@163.com; 2 School of Chemistry and Pharmaceutical Engineering, Jilin Institute of Chemical Technology, Jilin 132022, China; zfy18843226365@163.com (F.Z.); wangzhenlei2@jlict.edu.cn (Z.W.); yangwt_1981@163.com (X.Y.); 3 Peking Union Second Pharmaceutical Factory Ltd., Beijing 102600, China; zhangweina1992@yeah.net

**Keywords:** *Myristica fragrans* Houtt., processing, toxic components, antioxidant activity, antibacterial activity

## Abstract

This study aims to optimize the processing of *Myristica fragrans* Houtt. by talcum powder simmering using single-factor and orthogonal experimental methods, and the overall desirability values of dehydrodiisoeugenol and essential oils content were selected as indicators of the process. The new process reduced the total content of the three toxic components, namely myristicin, safrole and elemicin, from 1.91% to 1.16% before and after processing, indicating that the toxic components were reduced by 39%. The IC_50_ of the essential oils before and after processing were 1.002 ± 0.05 and 0.233 ± 0.05 mg/mL for DPPH scavenging activity and 0.132 ± 0.04 and 0.057 ± 0.05 mg/mL for ABTS scavenging activity, respectively. And the absorbance of the antioxidant activity against Ferric reducing power ranged from 0.213 to 0.709 and from 0.225 to 0.755, respectively. The minimum inhibitory concentration for *Staphylococcus aureus*, *Bacillus pumilus* and *Escherichia coli* were all lower after processing than before. The antioxidant activity and antibacterial activity of the essential oils after processing were better than before. The results of the survival of zebrafish embryos at different concentrations of essential oils at 0–168 h post fertilisation were higher after processing than before. These findings suggest that processing plays the role of reducing toxicity and increasing beneficial effects. They provide a scientific basis not only for the processing of *M. fragrans*, but also for the processing of other foods.

## 1. Introduction

*Myristica fragrans* Houtt. is the dried, mature seed kernel of nutmeg found in Malaysia, Indonesia, the West Indies and elsewhere [1]. *M. fragrans* is widely used as a domestic spice for cooking, in cakes and in drinks [2]. It is mainly used as a condiment owing to its unique flavour, colour or preservation effect [1]. *M. fragrans* contains a variety of functionally active components, such as essential oils, lignans, phenylpropanoids, total phenolics and flavonoids. It has functional activities such as anticancer, antioxidant, bacteriostatic, etc. [3].

Essential oils are among the main components of *M. fragrans* [4]. *M. fragrans* essential oils have the odour and taste of *M. fragrans* and can be used as a flavouring agent instead of *M. fragrans* powder. The essential oils are considered as promising food preservatives due to their proven antibacterial and antioxidant properties [5]. Both active and toxic components are present in *M. fragrans* essential oils. Three common toxic ingredients are myristicin, safrole and elemicin [6]. Myristicin can cause hepatic steatosis, leading to necrosis [7]. The toxic dose of myristicin is 1–2 mg, beyond which it may cause poisoning leading to many health problems associated with brain problems and, in severe cases, to death [8]. Safrole is both hallucinogenic and carcinogenic [1], but there are no clear consumption dose limits in the available studies. Elemicin exposure can cause hepatomegaly and hepatic ossification, and the levels of elemicin in food and spices are in the range of 1.2–150 mg/kg [9]. Abuse of *M. fragrans* as a psychoactive drug at doses of 3–5 g can lead to psychoactive effects, and the intake doses shall not exceed 20–25 g [6].

Lignans, one of the main functional components of *M. fragrans*, have the same functional activity as *M. fragrans* and are non-volatile [10]. They have antioxidant, bacteriostatic, anti-inflammatory, anti-cancer, anti-diabetes, liver and nerve protection, prevention of osteoporosis and other activities [11]. Among them, the lignan component dehydrodiisoeugenol is the active ingredient of *M. fragrans* and is chemically stable, with a strong antibacterial and inhibitory effect on intrahepatic lipid oxidation [12,13]. Due to its high content and activity, it is used as an evaluation indicator for the processing of *M. fragrans* [14].

People are now paying great attention to the nutritional hygiene and safety of food, and the presence of factors harmful to the human body in food will directly affect the safety of consumers. Food production and storage processes will produce a series of chemical changes; therefore, the choice of appropriate processing technology is important to improve food safety. There are many processes in food processing, such as frying, steaming, simmering and so on. *M. fragrans* implies other processing methods such as flour simmering, bran simmering, etc., and the alcohol extract of ·OH, O_2_^−^· and its scavenging capacity are lower than before processing, while its antioxidant activity is also reduced [15]. Moreover, during the bran simmering processing operation, burning can easily occur with the increase in temperature. Simmering is a widespread processing method, the main purpose of which is to remove some of the essential oils and irritating components, thus reducing the side effects [16]. After simmering, *M. fragrans* is brittle and fragile, making it easy to eat. In talcum powder simmering, talcum powder is used to absorb some of the volatile and irritating ingredients to reduce the side effects or moderate the medicinal properties and enhance their effect. Talcum powder simmering is also used for a variety of foods such as *Aucklandia lappa* Decne., *M. fragrans*, *Pueraria lobata* (Willd.) Ohwi and *Terminalia chebula* Retz. [17]. The contents of volatiles in *M. fragrans* are closely related to the processing temperature, time and excipients, which can reduce toxic substances and facilitate the consumption of foods.

This study investigated the *M. fragrans* simmering process for talcum powder from three aspects: talcum powder dosage, processing temperature and processing time. The overall desirability values of dehydrodiisoeugenol and essential oils content were selected as indicators of the process. To compare the changes in the chemical components, the antioxidant and antibacterial effects of *M. fragrans* essential oils before and after processing (marked OMB and OMA, respectively) were recorded. The changes in *M. fragrans* toxicity before and after processing were also assessed by zebrafish animal models. It is essential to develop a simple and efficient processing technique, especially for the *M. fragrans* flavour industry, in order to reduce toxicity and improve product quality. It may also serve as reference for the processing of other foods.

## 2. Results and Discussion

### 2.1. Single-Factor Experiment

The standard curve equation for dehydrodiisoeugenol was y = 1.1738x + 0.1319, R^2^ = 0.9999, and the linearity was well in the range of 1.875~60 µg/mL. And the content of dehydrodiisoeugenol before processing was 0.24%.

The effect of processing temperature within the range of 100–180 °C was studied with a processing time of 15 min and a talcum powder dosage of 50 g. The results show that the content of dehydrodiisoeugenol increased and then decreased with increasing temperature, and the content of OMA gradually decreased. Based on the highest overall desirability (OD) value of 79.17, a temperature of 140 °C was selected (No. 1–5 of Table 1).

The effect of processing time within the range of 10–30 min was studied at a processing temperature of 140 °C and a talcum powder dosage of 50 g. The dehydrodiisoeugenol content and OMA content increased and then decreased with increasing time. Based on the highest OD value of 83.33, the time of 20 min was selected (No. 6–10 of Table 1).

Talcum powder dosage in the range of 30–70 g was studied at a processing temperature of 140 °C and a processing time of 20 min. The dehydrodiisoeugenol content also increased and then decreased with increasing talcum powder dosage, while the effect on OMA content was not significant. Based on the highest OD value of 95.83, the talcum powder dosage of 40 g was selected (No. 11–15 of Table 1).

Therefore, the optimal processing temperature, processing time and talcum powder dosage were 140 °C, 20 min and 40 g, respectively.

### 2.2. Optimisation of Orthogonal Experiments

Combining the experimental results of a single factor, the orthogonal experimental design was carried out with three factors of processing temperature (A), processing time (B) and talcum powder dosage (C) as the independent variables, and the OD value of dehydrodiisoeugenol and OMA content were referred to as the evaluating indices to obtain the orthogonal experimental design and the results of the analyses (Table 2) and the analysis of variance (ANOVA) (Table 3).

Intuitive analysis shows that the results of factor A (processing temperature) are as follows: A_2_ > A_3_ > A_1_; for factor B (processing time), they are B_3_ > B_2_ > B_1_; and for factor C (talcum powder dosage), they are C_3_ > C_1_ > C_2_. From the intuitive analysis, the optimal concoction process was A_2_B_3_C_3_. The ANOVA shows that, although there is no significant difference, the order of influence of the factors on the experimental results is A > B > C, i.e., the processing temperature has the greatest influence on the results, followed by the processing time and then the talcum powder dosage.

Combined with the ANOVA and intuitive analysis of orthogonal tests, the optimal processing conditions were A_2_B_3_C_3_. The processing temperature was 140 °C, the processing time was 25 min and the talcum powder dosage was 50 g. The final optimum processing parameters were determined as follows: processing temperature of 140 °C, processing time of 25 min and talcum powder dosage of 50%. The mean value of processed dehydrodiisoeugenol content was 0.25% with a relative standard deviation (RSD) of 2.16%, and the mean value of OMA yield was 6.43% with an RSD of 2.50%.

The present study showed a significant reduction in the content of essential oils at a temperature of 140 °C, which is lower than that found in the literature at 170 °C compared to the talcum powder stir-frying processing technique [14], which was of 6.9% in the literature and of 6.43% in the present study’s processing process. This further suggests that talcum powder simmering could adsorb volatile and irritant components, thereby reducing the content of toxic components. Dong et al. measured the average content of dehydrodiisoeugenol in the flour simmering process to be 0.27%, but it was necessary to peel the skin by hand, and when peeling the flour, the flour could easily stick to *M. fragrans*, which was not easy to handle, and at the same time, there was less exudation of the oil substance [18].

### 2.3. Scanning Electron Microscopy (SEM)

**Figure 1:**
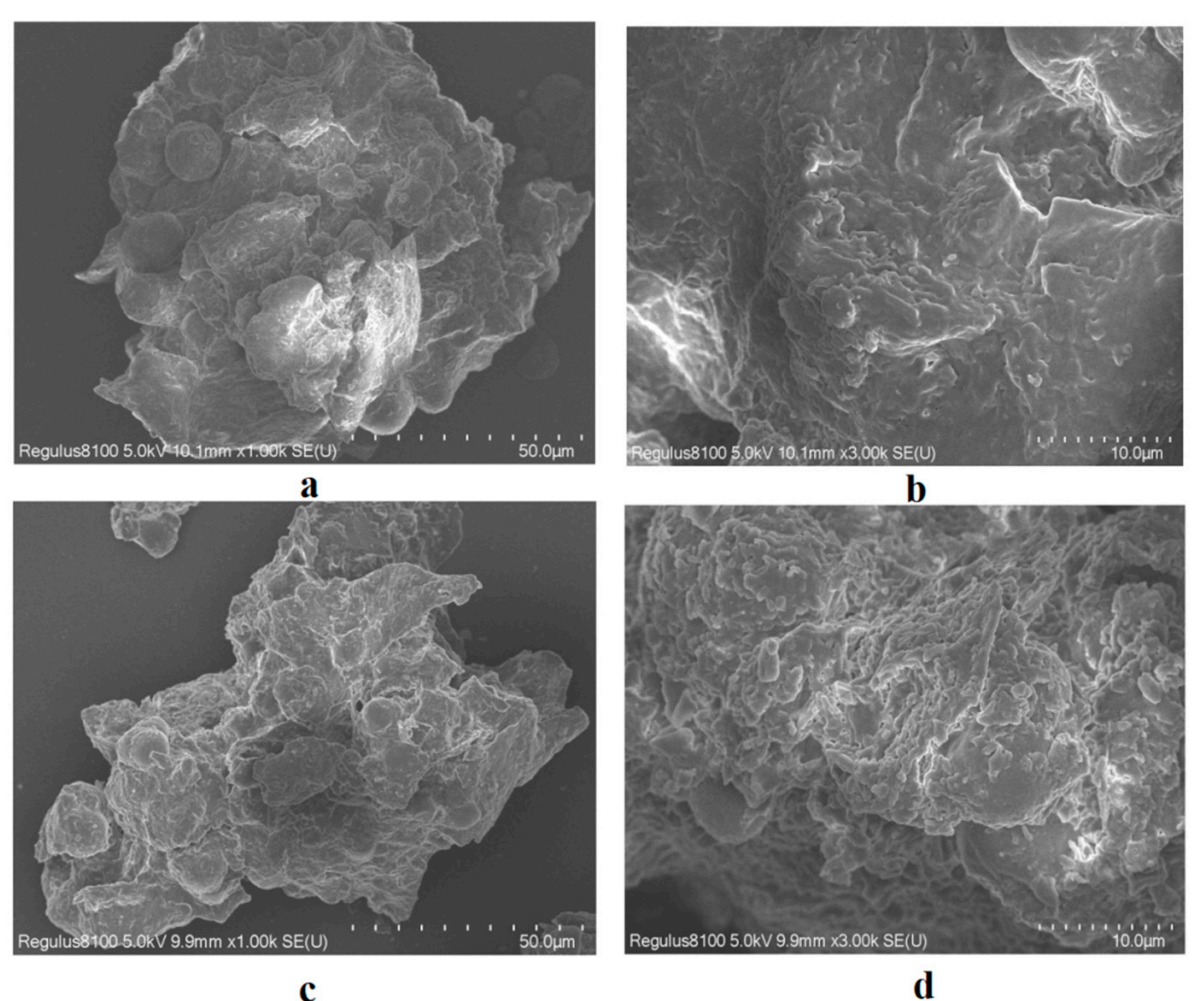
SEM images of the *M. fragrans* powder before and after processing. (**a**) Images of *M. fragrans* powder at 1000 magnification. (**b**) Images of *M. fragrans* powder at 3000 magnification. (**c**) Images of *M. fragrans* powder after processing at 1000 magnification. (**d**) Images of the *M. fragrans* powder after processing at 3000 magnification.

The SEM image shows that the unprocessed *M. fragrans* is relatively smooth and intact (Figure 1a,b). Starch granules were mostly single granules, with a few complex granules composed of 2–6 fractions, and they showed evident umbilical dots. The degree of crushing and deformation of the powder was enhanced after processing for *M. fragrans* (Figure 1c,d), which may be due to the high processing temperature.

### 2.4. Gas Chromatography–Mass Spectrometry (GC-MS)

Twenty-eight compounds were identified from OMB, accounting for 99.44% of the total. The most abundant compound was fenchlorphos (21.23%), followed by methyleugenol (15.98%), myristicin (12.95%) and elemicin (8.29%). Twenty-one compounds from OMA were identified, accounting for 99.76% of the total. The most abundant compound was fenchlorphos (26.23%), followed by myristicin (15.9%), α-pinene (14.82%) and β-pinene (10.3%).

The contents of toxic safrole and elemicin were reduced by 0.11% and 5.96%, respectively. The relative content of myristicin in OMA (15.9%) was higher than that in OMB (12.95%) (Table 4). This difference may be attributed to two reasons. First, the boiling points of safrole and elemicin are 232 °C and 146 °C, respectively, which are below the temperature of the gasification chamber of 250 °C and are more volatile. Myristicin has a boiling point of 276 °C, which is higher than the temperature of that the gasification chamber, and volatilises relatively little. Second, the relatively few constituents isolated and identified from OMA resulted in a little higher relative amount of myristicin. Importantly, the total content of the three toxic components, namely myristicin, safrole and elemicin, decreased from 1.91% to 1.16% relative to the total *M. fragrans* content before and after processing, indicating that the toxic components were reduced by 39%. Talcum powder is an excipient that absorbs oils and can adhere to the volatile and irritating components from the surface of *M. fragrans* after processing, so the content of myristicin, safrole and elemicin decreases after sieving [16].

Fenchlorophos is a widely used organophosphorus pesticide with a relatively high boiling point. It is a residue of the pesticide during the growth of *M. fragrans* [19]. The content of active ingredients such as α-pinene and β-pinene increased by 92% and 35%, respectively. The boiling point of methyl eugenol is 254 °C and the decrease in content may be due to a longer processing time and higher processing temperature [20]. The results show a reduction in the content of the toxic components of essential oils after processing.

### 2.5. Fourier Transform Infrared Spectrometry (FT-IR)

**Figure 2:**
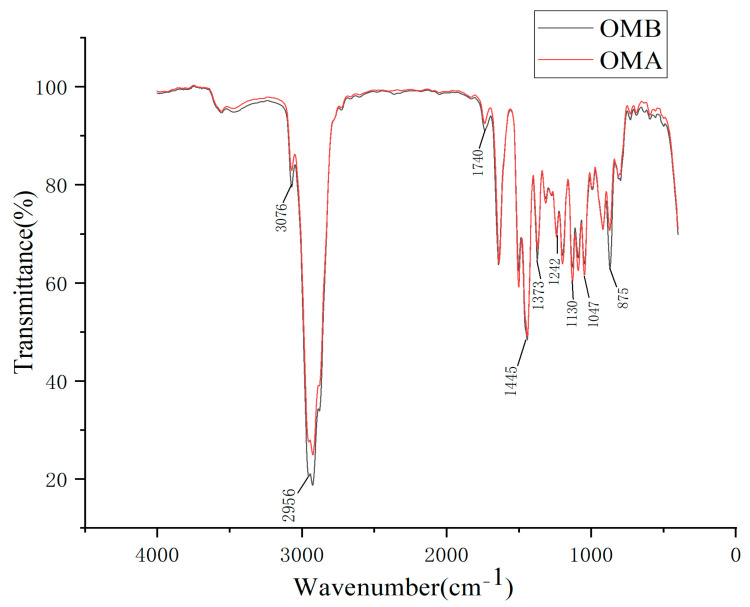
IR spectra of OMB and OMA.

Figure 2 shows the FTIR diagrams of OMB and OMA. The correlation coefficients of wavenumber at each point in the two infrared spectra calculated on SPSS24.0 were all 1. The two types of essential oils have the same functional groups.

The peak at 3076 cm^−1^ is the C-H stretching vibration of alkenes. The peaks at 1373 and 2956 cm^−1^ are the stretching vibration of saturated C-H in -CH_3_. The peak at 1250 cm^−1^ may be due to the symmetric expansion of the C-O-C of the aromatic acid ester and vibrational stretching of the C-OH group of the phenolic component, whereas the peak at 1445 cm^−1^ represents the vibrational bending absorption of C-OH by the alcohol moieties [21]. The peak at 1130 cm^−1^ is one strong peak of aliphatic ether. The peak at 1047 cm^−1^ is the R-O stretching vibration of aromatic ether [22]. The peak at 875 cm^−1^ is the C-H out-of-plane bending vibration absorption of aromatic hydrocarbons [23].

The main functional groups in Figure 2 are basically consistent with the compounds in Table 4, and the peak shapes of the two spectrograms before and after processing are basically the same. These results show that the processing changes the content of the essential oils, while the type of chemical composition does not change much.

### 2.6. Effect of Processing on Antioxidant Activity

**Figure 3:**
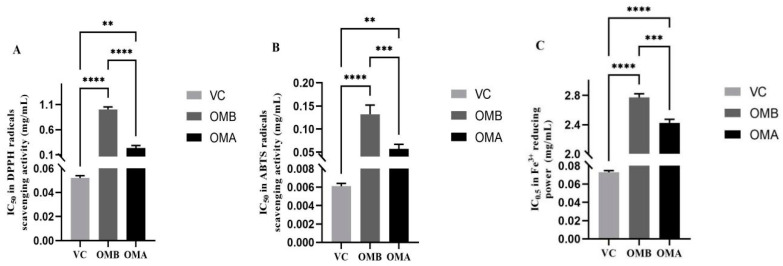
(**A**–**C**) represent the antioxidant capacities of OMB and OMA against DPPH radicals, ABTS·^+^ and Ferric reducing antioxidant power (FRAP), respectively. The significance of the difference between the two sets of data is represented as ** *p* < 0.01, *** *p* < 0.001, **** *p* < 0.0001, respectively.

There was a dose–effect relationship between OMB and OMA on the scavenging of 2,2-Diphenyl-1-picrylhydrazyl (DPPH) radicals in the range of 0.1–0.8 mg/mL (Figure 3A). In the range of 0.01–0.4 mg/mL, there was a certain dose–effect relationship on the scavenging rate of 2,2′-azinobis-(3-ethylbenzthiazoline-6-sulphonate) (ABTS) radicals (Figure 3B). The IC_50_ of OMB and OMA against DPPH radicals scavenging activity was 1.002 ± 0.05 and 0.233 ± 0.05 mg/mL, respectively, and that against ABTS·^+^ scavenging activity was 0.132 ± 0.04 and 0.057± 0.05 mg/mL, respectively, indicating that OMA has higher antioxidant activity than OMB. The IC_50_ for Vitamin C (Vc) in DPPH was 0.052 ± 0.002 mg/mL and for ABTS it was 0.006 ± 0.0003 mg/mL. In the concentration range from 1 to 6 mg/mL, some reducing power was observed for Fe^3+^ (Figure 3C). The absorbance of OMB was 0.213~0.709 ± 0.03, and the absorbance of OMA was 0.225~0.755 ± 0.02. The absorbance of OMA was higher than that of OMB over the concentration range. In a word, OMA showed higher antioxidant capacity than OMB in all three systems.

The enhanced antioxidant effect of OMA may be attributed to the increased contents of α-phellandrene and myrcene. Although myristicin and elemicin have antioxidant effects [24], these effects have not been affected by their reduced contents. This indicates that there is a synergy between the chemical components.

### 2.7. Effect of Processing on Antibacterial Activity

The results of essential oils against all strains of microorganisms tested are shown in Table 5. The minimum inhibitory concentration (MIC) of OMA against *Staphylococcus aureus*, *Bacillus pumilus* and *Escherichia coli* were all lower than for OMB (Table 5), indicating that OMA has enhanced bacteriostatic activity. No bacteriostatic activity was measured against *Saccharomyces cerevisiae* or *Candida albican*.

Essential oils contain antioxidant and bacteriostatic constituents, including α-phellandrene and β-myrcene [25]. α-Pinene and β-pinene have an antibacterial effect on *Escherichia coli*, *Staphylococcus aureus* and other microorganisms, and their antibacterial abilities are improved with the increase in dosage [26]. Elemicin possesses strong antibacterial effects [9]. Other chemical components may also have a synergistic effect.

With the reduction in elemicin contents in OMA, the antioxidant and antimicrobial effects are improved, indicating that processing plays a role in food safety and that the optimised process could be used in food processing.

### 2.8. Zebrafish Embryo Upgrowth Toxicity

The survival rates of zebrafish embryos at different concentrations (1.5, 3, 6, 12, 25 and 50 µg/mL) under 0–168 h post fertilisation (hpf) conditions are shown in Table 6. At the lower concentrations of 1.5 and 3 µg/mL, there was no significant decrease in survival and OMA showed a high survival rate of up to 90%. At 6, 12 and 25 ug/mL for 96 hpf, the survival rates were 50%, 50% and 40% for OMB and 70%, 70% and 60% for OMA, respectively, which were significantly different. At 50 µg/mL, the survival rate dropped significantly to 20% at 48, 72 and 96 hpf for OMB, respectively, and the *p*-value before and after processing was less than 0.05, which was significant. The survival rate of OMA was higher than that of OMB after 96 hpf at different concentrations, and both were significantly different. The results of the survival rates are as follows: control > OMA > OMB. The survival rate of OMB was significantly lower than that of OMA.

It can be concluded that, in agreement with the results of GC-MS and IR analysis, the toxicity of OMA was reduced and the simmering method had a reducing effect on toxicity. The main reason for this is that the talcum powder in the simmering process can adhere to volatile and irritating components such as safrole and elemicin, and therefore its toxic content decreases after sieving.

## 3. Material and Methods

### 3.1. Materials and Reagents

*M. fragrans* was purchased from Hebei Golden Leaf Pharmaceutical Co., Ltd. (LOT: 200501C159). The materials were identified by Prof. Xiudong Yang (Jilin Institute of Chemical Technology, Jilin, China) and determined as *Myristica fragrans* Houtt. 2,2-Diphenyl-1-picrylhydrazyl (DPPH) and 2,2′-azinobis-(3-ethylbenzthiazoline-6-sulphonate) (ABTS) were provided by TCI Chemical Industry Development Co., Ltd., Shanghai, China. Wild-type (AB line) zebrafish were purchased from the Shandong YiXiYue Biotechnology Co., Ltd., Shandong, China. All reagents were of analytical grade.

### 3.2. Simmering M. fragrans with Talcum Powder

The stir-frying machine was raised to the desired temperature. Then, the talcum powder was poured in and simmered until it became flexible (about 3 min). Next, the whole *M. fragrans* was placed in the machine and simmered for the desired time. Then, it was removed rapidly, and the talcum powder was sieved, cooled to room temperature and placed in a dry bag.

### 3.3. Detection of Dehydrodiisoeugenol

Take 3.0 mg of dehydrodiisoeugenol standard and methanol as solvent. The standard solution of dehydrodiisoeugenol was prepared as 1.875, 3.75, 7.5, 15, 30 and 60 µg/mL, and the standard curve was plotted with the peak area as the vertical coordinate and the concentration of the control as the horizontal coordinate. The processed *M. fragrans* was ground into powder and passed through a 20-mesh sieve. The powder (0.5 g) was placed into a conical flask with a stopper and added to 50 mL of methanol under ultrasonic treatment (250 W, 30 kHz) for 30 min. After cooling, weigh again, and make up the lost weight with methanol, shake well, filter and take the filtrate as the sample solution.

The standard and sample solution were filtered through a 0.45 μm membrane, while 20 μL of filtrate was injected into the Ultimate 3000 HPLC (Thermo, New York, NY, USA) equipped with an Ultimate 3000 diode array detector (DAD, Thermo) and a Inertsil ODS-3 column (5 μm, 4.6 × 250 mm). The mobile phase was methanol and water (75:25) with the elution. The flow rate was 1.0 mL/min and the column temperature was 25 °C. The detection wavelength was 274 nm. The content of dehydrodiisoeugenol is calculated according to Equation (1):

Content of dehydrodiisoeugenol (%) = [(C × V × 10^−3^)/M] × 100% (1)

where C is the concentration of dehydrodiisoeugenol, V is the dilution ratio of the solution and M is the mass of *M. fragrans* powder.

### 3.4. Extraction of M. fragrans Essential Oils

*M. fragrans* essential oils were extracted for 5 h through steam distillation with 20 g of powder and 300 mL of water under the processing temperature of 140 °C, the processing time of 25 min and the talcum powder dosage of 50 g. The obtained oils were dried with anhydrous sodium sulfate and stored at 4 °C until analysis. The essential oil yield was calculated according to Equation (2):

Yield (%) = (volume of essential oils/weight of crude drug) × 100% (mL/g)(2)

### 3.5. Calculation of the Composite Weighted Score

In order to comprehensively investigate the influence of the processing conditions, the dehydrodiisoeugenol and essential oils content were assigned their weights, and the test results of multiple indicators were converted into single indicators through weighted sum, which not only avoided the limitations and inaccuracies caused by the evaluation of a single factor, but also took into account the influence on multiple indicators, so as to make the optimisation of the process more scientific and rationalised. Dehydrodiisoeugenol is the active ingredient of *M. fragrans* and is chemically stable, the essential oils contain toxic components and the temperature and time of processing have an effect on the essential oils’ content and vary greatly. The coefficients were set to 1 for essential oils and 3 for dehydrodiisoeugenol in order to make the effect of essential oils on the experimental results small. Dehydrodiisoeugenol content (75%) and essential oils content (25%) were used as evaluation indices for analysis. A comprehensive weighted scoring method was used for scoring, and the scoring index for dehydrodiisoeugenol content was Y_1_, for essential oils content it was Y_2_ and the maximum OD value was the optimal processing condition.

Y_1_ = (Y_i_ − Y_min_)/(Y_max_ − Y_min_) × 75 Y_2_ = (Y_max_ − Y_i_)/(Y_max_ − Y_min_) × 25 Y = Y_1_ + Y_2_ (3)

where Y_min_ is the minimum value in the indicator, Y_max_ is the maximum value in the indicator and the OD value is Y.

### 3.6. Single-Factor Experiments

Three factors including processing time (10, 15, 20, 25 and 30 min), processing temperature (100, 120, 140, 160 and 180 °C) and talcum powder dosage (30, 40, 50, 60 and 70 g), each at five levels, were selected. In a single-factor experiment, only one factor was changed while the other two were unchanged. The content of dehydrodiisoeugenol and essential oil was determined for each serving of 100 g *M. fragrans* and the OD was calculated.

### 3.7. Optimisation of Orthogonal Experiments

The statistical analysis software Orthogonal Design Assistant V 3.1 was used to design the orthogonal experiments. A three-factor, three-level experiment was designed using processing temperature, processing time and talcum powder dosage as independent variables and OD values of dehydrodiisoeugenol and volatile oil scores as evaluation indicators.

### 3.8. SEM

SEM allows for observation of the micro-morphological characterisation of substances and allows for a more intuitive comparison of the effects of processing on them. The *M. fragrans* powder before and after processing was observed by SEM [27]. The sieved powder with an internal diameter of 850 ± 29 μm was gold-sprayed for 120 s, and then examined by SEM (Regulus 8100, Hitachi, Tokyo, Japan) at an acceleration voltage of 5.0 kV (500–3000 magnification).

### 3.9. FT-IR of Essential Oils

FT-IR allows for the observation of functional groups in the structure of essential oils and for a further comparison of the changes in the composition of essential oils before and after processing. The IR spectra of OMB and OMA were obtained using an FT-IR meter (Gangdong SCI. & TECH. Development Co. Ltd., Tianjin, China). First, OMB or OMA (50 μL) was processed and covered evenly to form a thin layer. Then, infrared spectra were measured in the range of 500–4000 cm^−1^. The instrument resolution was 0.74 cm^−1^, scanned 32 times. SPSS24.0 was used to calculate the wavenumber correlation coefficient of each spot in the two IR spectra.

### 3.10. GC-MS of Essential Oils

GC-MS technology allows for an accurate analysis of the composition of essential oils and comparison of changes in the composition of essential oils before and after processing. The presence of OMB and OMA was investigated by GC-MS (Shimadzu GC-MS-2010-plus, shimadzu, Kyoto, Japan) [28]. The chromatographic conditions included an Rxi 5MS capillary column (30 m, 0.25 mm, 0.25 µm) with helium as carrier gas at 1 mL/min, and a sample volume of 1.0 μL. The GC oven was kept at 6 °C for 6 min, adjusted to 250 °C at a rate of 3 °C/min and then kept constant for 30 min. The injection port temperature was 250 °C and the column pressure was 49.5 kPa. For MS, an electron ionisation source at 230 °C was used. The electron energy was 70 eV and the scanning mass range was 40–500 *m*/*z*. The essential oils were diluted 10 times with methanol and filtered through microporous membrane for GC-MS. Constituents were identified from mass spectra and retention time using the NIST library. The relative content of each constituent (%) was calculated based on the peak area of GC.

### 3.11. Antioxidant Activity of Essential Oils

Three parallel experiments were performed for each sample, and the average value was taken. Vitamin C was used as a positive control. The concentration of a sample that inhibited 50% of the radical (IC_50_) was calculated.

DPPH radical scavenging activity was determined according to the method of Wang et al. [27]. The essential oils were dissolved in ethanol, and a series of solutions at different concentrations (0.1–0.8 mg/mL) were conducted. The absorbance was measured with a UV spectrometer at 517 nm. The ethanol was used as a blank.

ABTS·^+^ scavenging activity was determined according to the method of Li et al. [28]. The essential oils were dissolved in absolute ethanol, and a series of solutions at different concentrations (0.01–0.4 mg/mL) were made. Then, the absorbance at 734 nm was measured and ABTS (2.0 mL) plus 2.0 mL of absolute ethanol was used as a control.

FRAP was measured according to JD Adámez et al. [29]. The essential oils were dissolved in absolute ethanol, and a series of solutions at different concentrations (0.5–5 mg/mL) were made. The absorbance at 700 nm was detected.

### 3.12. Antimicrobial Activity of Essential Oils

The MIC was detected using a broth microdilution assay [28]. All the bacterial strains, including *Staphylococcus aureus* ATCC 29213, *Saccharomyces cerevisiae* ATCC 27457, *Bacillus pumilus* ATCC 700814, *Escherichia coli* ATCC 25922 and *Candida albican* ATCC 10231, were bought from Beijing Yuding Xinjie Technology Co., Beijing, China. Samples of 5 concentrations (15.625–500 µg/mL) were made with 20% dimethyl sulfoxide (DMSO) solution as the solvent. In a 96-well plate, 180 µL of a bacterial standard suspension (10^8^ CFU/mL) and 20 µL of a sample solution were added to each well. The sample concentration in the following wells was reduced by serial dilution 2-fold. Chloramphenicol was used as a positive control and 20% DMSO solution without essential oils was used as a negative control.

### 3.13. Assessment of Zebrafish Embryo Upgrowth Toxicity

All adult zebrafish were kept in rearing conditions (28 °C, pH 7.8–8.0, 14/10 h light/dark cycle) in a rearing facility [30]. Zebrafish were fed twice daily. Male and female zebrafish were placed in the incubator in a 2:1 ratio, with males and females separated by a partition, which was withdrawn the following day, and fertilised eggs were obtained the next morning 14 h later. Embryos were obtained from natural spawning induced by turning on the light in the morning. Embryos were washed and disinfected with 0.01% (*v*/*v*) methylene blue solution for breeding.

Healthy embryos were selected under a microscope at 48 hpf and 10 embryos per well were randomly placed in a sterile 6-well plate. A solvent control (0.04% DMSO) and a sample solution for each group (concentration gradient of 1.5, 3, 6, 12, 25 and 50 μg/mL) was added to each well in a final volume of 2 mL. The exposed solution was replaced after 24 h. At the same time, dead embryos were removed and mortality was recorded. The number of embryos surviving at different time points after administration was then recorded at 24, 48, 72, 96, 120, 144 and 168 hpf, respectively (repeated experiments, *n* = 3).

### 3.14. Statistical Analysis

Experimental data were statistically tested by using SPSS24.0. One-way analysis of variance (ANOVA) was used by GraphPad Prism 9.0. All assays were conducted in triplicate. Results were expressed as means ± SD. The main effects were the samples before and after processing as well as the selected concentration, and the random effects were indicators that the experiment was repeated.

## 4. Conclusions

In this study, the processing technology of *M. fragrans* by simmering was optimised with the overall desirability values of dehydrodiisoeugenol and essential oils content as evaluation indices and talcum powder as excipient. This is the first study to investigate the changes in the chemical components and activity of *M. fragrans* during processing. The results show that processing reduces the content of toxic components from UV, IR and GC-MS analyses, which has not significantly changed the chemical composition category of the essential oils. The talcum powder simmering process reduces the content of myristicin, safrole and elemicin, which are harmful components of essential oils, and improves their antioxidant and antibacterial activities. This result illustrates that processing plays a role in reducing toxicity and increasing beneficial effects. These findings will provide some scientific basis for further research on *M. fragrans* and a new method in relation to processes related to toxic ingredients in foods. The selection of appropriate processing techniques has made it possible to develop many new functional foods, expanding the range of products currently produced and increasing their safety and functionality, which is important for human health and improving economic development.

## Figures and Tables

**Table 1:** Results of single-factor experiments for *M. fragrans* by talcum powder simmering.

No.	Processing Time (Min)	Processing Temperature (°C)	Talcum Powder Dosage (%)	Dehydrodiisoeugenol Content (%)	OMA Yield (%)	OD
1	15	100	50	0.225	7.00	69.00
2	15	120	50	0.226	6.90	73.67
3	15	140	50	0.227	6.75	79.17
4	15	160	50	0.218	6.90	49.67
5	15	180	50	0.202	5.50	25.00
6	10	140	50	0.150	7.00	20.09
7	15	140	50	0.190	7.50	27.97
8	20	140	50	0.306	7.00	83.33
9	25	140	50	0.212	6.50	53.56
10	30	140	50	0.121	6.00	25.00
11	20	140	30	0.289	8.00	82.92
12	20	140	40	0.290	7.25	95.83
13	20	140	50	0.271	7.00	92.13
14	20	140	60	0.120	8.50	4.56
15	20	140	70	0.109	8.00	8.33

**Table 2:** Results and analysis of orthogonal experiments for *M. fragrans* by talcum powder simmering.

No.	Processing Temperature (°C)	Processing Time (min)	Talcum Powder Dosage (%)	Y_1_	Y_2_	OD
1	120	15	30	10.15	25.00	35.15
2	120	20	40	19.74	14.29	34.02
3	120	25	50	36.65	0.00	36.65
4	140	15	40	18.05	7.14	25.19
5	140	20	50	75.00	17.86	92.86
6	140	25	30	64.85	21.43	86.28
7	160	15	50	23.12	17.86	40.98
8	160	20	30	0.00	7.14	7.14
9	160	25	40	41.17	17.86	59.02
K_1_	35.273	33.773	42.857			
K_2_	68.110	44.673	39.410			
K_3_	35.713	60.650	56.830			
R	32.837	26.877	17.420			

Y_1_: the scoring index for dehydrodiisoeugenol content. Y_2_: the scoring index for essential oils content.

**Table 3:** Orthogonal experiments ANOVA for *M. fragrans* by talcum powder simmering.

Factor	Sum of Squared Deviations	Degrees of Freedom	*F* Value	*p* Value
A	2127.984	2	1.350	0.350
B	1096.419	2	0.696	0.590
C	510.59	2	0.324	0.755
Empty (error)	2589.12	2		

**Table 4:** Constituents of OMB and OMA.

No.	Compound	Molecular Formula	RI ^a^	RI ^b^	Content, %	
			Calculated Value	Reference Value	OMB	OMA
1	3-Thujene	C_10_H_16_	902	902	0.87	1.8
2	β-Pinene	C_10_H_16_	930	948	7.61	10.3
3	(1S)-(+)-3-Carene	C_10_H_16_	940	948	2.18	3.48
4	3-Carene	C_10_H_16_	944	948	0.25	0.21
5	Myrcene	C_10_H_16_	945	958	1.39	1.64
6	α-Pinene	C_10_H_16_	948	948	7.7	14.82
7	3-Methylene-6-(1-methylethyl)-Cyclohexene	C_10_H_16_	961	964	0.04	-
8	Camphene	C_10_H_16_	958	943	5.16	7.72
9	α-Phellandrene	C_10_H_16_	965	969	0.34	0.5
10	Fenchlorphos	C_10_H_16_	991	993	21.23	26.23
11	Terpinene	C_10_H_16_	987	998	0.05	-
12	Sabinene	C_10_H_16_	1029	1030	0.08	0.04
13	Terpinolene	C_10_H_16_	1049	1052	1.24	2.02
14	1-Methyl-2-(1-methylethyl)benzene	C_10_H_16_	1035	1042	0.25	0.49
15	Terpinen-4-ol	C_10_H_18_O	1136	1137	4.84	3.77
16	α-Terpineol	C_10_H_18_O	1138	1143	0.2	0.11
17	Linalyl acetate	C_12_H_20_O_2_	1267	1272	0.06	-
18	Bornyl acetate	C_12_H_20_O_2_	1271	1277	0.05	0.03
19	Safrole	C_10_H_10_O_2_	1319	1327	0.35	0.24
20	α-Cubebene	C_15_H_24_	1326	1344	0.12	-
21	Methylisoeugenol	C_11_H_14_O_2_	1334	1361	6.57	-
22	e-β-Farnesene	C_15_H_24_	1339	1440	-	0.06
23	Methyleugenol	C_12_H_20_O_2_	1358	1361	15.98	7.9
24	Farnesene	C_15_H_24_	1424	1458	0.03	-
25	α-Muurolene	C_15_H_24_	1428	1440	0.19	-
26	β-Caryophyllene	C_15_H_24_	1490	1494	0.27	0.17
27	Myristicin	C_11_H_12_O_3_	1508	1516	12.95	15.9
28	Elemicin	C_12_H_16_O_3_	1544	1550	8.29	2.33
29	Myristic acid	C_14_H_28_O_2_	1764	1769	1.15	-

^a^ RI: retention index (RI) relative to standard mixture of n-alkanes on Rxi 5MS column. ^b^ RI: RI reference values retrieved from the National Institute of Standards and Technology (NIST) mass spectrometry library.

**Table 5:** MICs of OMB and OMA against three microbial strains.

Name	*S. aureus*	*B. pumilus*	*E. coli*
OMB (µg/mL)	250 ± 0.05 ^a^	250 ± 0.06 ^a^	250 ± 0.05 ^a^
OMA (µg/mL)	31.25 ± 0.03 ^b^	62.5 ± 0.05 ^b^	250 ± 0.04 ^a^
Chloramphenicol (µg/mL)	6.25 ± 0.02 ^c^	3.13 ± 0.04 ^c^	3.13 ± 0.02 ^b^

Values are presented as mean ± SD. ^a,b,c^ Means in the same microbial strains with different superscripts are significantly different (*p* < 0.05).

**Table 6:** Determination of the effect of different concentrations of OMB and OMA on the survival of zebrafish embryos.

Concentration (µg/mL)	Name	0 hpf	48 hpf	72 hpf	96 hpf	120 hpf	144 hpf	168 hpf
1.5	OMB	100 ± 0.0 ^a^	100 ± 0.0 ^a^	100 ± 0.0 ^a^	90 ± 5.8 ^a^	90 ± 5.8 ^a^	80 ± 5.8 ^a^	80 ± 10.0 ^a^
	OMA	100 ± 0.0 ^a^	100 ± 0.0 ^a^	100 ± 0.0 ^a^	100 ± 0.0 ^b^	90 ± 5.8 ^a^	90 ± 5.8 ^b^	90 ± 5.8 ^b^
3.0	OMB	100 ± 0.0 ^a^	100 ± 0.0 ^a^	100 ± 0.0 ^a^	90 ± 5.8 ^a^	80 ± 5.8 ^a^	70 ± 5.8 ^a^	70 ± 5.8 ^a^
	OMA	100 ± 0.0 ^a^	100 ± 0.0 ^a^	100 ± 0.0 ^a^	100 ± 0.0 ^b^	90 ± 5.8 ^b^	80 ± 10.0 ^b^	80 ± 5.8 ^b^
6.0	OMB	100 ± 0.0 ^a^	90 ± 5.8 ^a^	90 ± 5.8 ^a^	80 ± 5.8 ^a^	80 ± 5.8 ^a^	70 ± 10.0 ^a^	50 ± 10.0 ^a^
	OMA	100 ± 0.0 ^a^	100 ± 0.0 ^b^	100 ± 0.0 ^b^	90 ± 5.8 ^b^	90 ± 5.8 ^b^	80 ± 10.0 ^b^	70 ± 10.0 ^b^
12.0	OMB	100 ± 0.0 ^a^	90 ± 5.8 ^a^	90 ± 5.8 ^a^	80 ± 5.8 ^a^	70 ± 10.0 ^a^	60 ± 5.8 ^a^	50 ± 10.0 ^a^
	OMA	100 ± 0.0 ^a^	100 ± 0.0 ^b^	90 ± 5.8 ^a^	90 ± 5.8 ^b^	80 ± 10.0 ^b^	80 ± 5.8 ^b^	70 ± 5.8 ^b^
25.0	OMB	100 ± 0.0 ^a^	90 ± 5.8 ^a^	80 ± 5.8 ^a^	80 ± 5.8 ^a^	70 ± 10.0 ^a^	50 ± 10.0 ^a^	40 ± 15.2 ^a^
	OMA	100 ± 0.0 ^a^	100 ± 0.0 ^b^	90 ± 5.8 ^b^	90 ± 5.8 ^b^	80 ± 5.8 ^b^	70 ± 5.8 ^b^	60 ± 10.0 ^b^
50.0	OMB	100 ± 0.0 ^a^	60 ± 5.8 ^a^	40 ± 10.0 ^a^	20 ± 10.0 ^a^	20 ± 10.0 ^a^	10 ± 10.0 ^a^	10 ± 10.0 ^a^
	OMA	100 ± 0.0 ^a^	80 ± 5.8 ^b^	70 ± 5.8 ^b^	50 ± 5.8 ^b^	40 ± 10.0 ^b^	20 ± 10.0 ^b^	20 ± 10.0 ^b^
-	Control (0.04%DMSO)	100 ± 0.0	100 ± 0.0	100 ± 0.0	100 ± 0.0	100 ± 0.0	100 ± 0.0	100 ± 0.0

Values are presented as mean ± SD. ^a,b^ Means in the same concentration and time with different superscripts are significantly different (*p* < 0.05).

## Data Availability

Data are contained within the article.
